# Unraveling the Impact of KRAS Accessory Proteins on Oncogenic Signaling Pathways

**DOI:** 10.3390/cells15020190

**Published:** 2026-01-20

**Authors:** Vanshika Garg, Raphael N. H. M. Hofmann, Moazzam Saleem, Amin Mirzaiebadizi, Ghazaleh Sadat Hashemi, Tooba Hameed, Bahareh Jooyeh, Silke Pudewell, Mehrnaz Mehrabipour, Niloufar Mosaddeghzadeh, Roland P. Piekorz, Mohammad Reza Ahmadian

**Affiliations:** Institute of Biochemistry and Molecular Biology II, Medical Faculty, Heinrich Heine University Düsseldorf, Universitätsstrasse 1, Building 22.03, 40225 Düsseldorf, Germany; vanshika.garg@hhu.de (V.G.); raphael.hofmann@uni-duesseldorf.de (R.N.H.M.H.); moazzam.saleem@hhu.de (M.S.); amin.mirzaiebadizi@hhu.de (A.M.); ghazalehsadat.hashemi@studenti.unimi.it (G.S.H.); tuba.hameed10@gmail.com (T.H.); b.jooyeh@gmail.com (B.J.); silke.pudewell@hhu.de (S.P.); mehrnaz.mehrabipour@childrens.harvard.edu (M.M.); niloufar.mosaddeghzadeh@uk-koeln.de (N.M.); roland.piekorz@hhu.de (R.P.P.)

**Keywords:** accessory proteins, KRAS oncogene, adenocarcinoma, Galectin-3, PDE-delta, nucleophosmin, IQGAP1, SHOC2, MAPK, AKT, signal transduction

## Abstract

**Highlights:**

**What are the main findings?**

- Target Potency: Knocking out *GAL3* and *PDEδ* significantly impaired MAPK signaling and reduced AKT-related signaling. GAL3 primarily impacted the mTORC2-AKT pathway, and PDEδ inhibited the mTORC2-AKT and PI3K-AKT pathways. This led to a substantial reduction in cancer cell proliferation.
- Pathway Specificity: SHOC2 selectively disrupted the MAPK pathway, and IQGAP1 knockout increased PI3K-AKT signaling. These results demonstrate that these accessory proteins have distinct, non-redundant roles in KRAS regulation.

**What is the implication of the main finding?**

- New Therapeutic Avenues: GAL3 and PDEδ are promising candidates for combinatorial drug development and could overcome the limitations of current direct KRAS inhibitors.
- Overcoming Resistance: Targeting these modulators could suppress the compensatory signaling that contributes to resistance to KRAS-targeted therapies.

**Abstract:**

The oncogene KRAS drives tumor growth by activating pathways such as MAPK and PI3K-AKT in a constitutive manner. Although direct KRAS inhibitors exist, they are often limited in clinical use due to therapeutic resistance and toxicity. Therefore, alternative combinatorial therapeutic strategies are urgently needed. This study examined the knockout of five KRAS-related proteins—galectin-3 (GAL3), phosphodiesterase delta (PDEδ), nucleophosmin (NPM1), IQ motif-containing GTPase-activating protein 1 (IQGAP1), and SHOC2—using CRISPR-Cas9 in adenocarcinoma cell lines harboring the KRAS(G12V) oncogenic mutation, as well as in the noncancerous HEK-293 cell line. These proteins act as critical modulators that regulate KRAS activity, cellular localization, and that of its downstream signaling components. We analyzed the downstream activation of ERK and AKT kinases and evaluated subsequent cancer cell proliferation. Knockout of *GAL3* and *PDEδ* was highly effective, significantly reducing MAPK and PI3K-AKT pathway activity and substantially impairing cell proliferation. *SHOC2* knockout selectively and potently disrupted MAPK activation, while NPM1 knockout resulted in the complex, reciprocal modulation of the two major pathways. Notably, knocking out *IQGAP1* enhanced PI3K–AKT and mTORC2–AKT signaling without affecting the MAPK pathway. These distinct modulatory roles highlight the non-redundant functions of the accessory proteins. In conclusion, our findings establish GAL3 and PDEδ, two KRAS-associated proteins, as promising combinatorial drug targets. Targeting these modulators provides an effective alternative strategy to overcome resistance mechanisms and enhance the clinical utility of existing KRAS inhibitors.

## 1. Introduction

KRAS4B (hereafter referred to as KRAS) plays a central role in the transduction of signals from receptor tyrosine kinases to intracellular effectors involved in several signaling pathways, particularly those regulating cell survival and proliferation. KRAS functions as a molecular switch by cycling between an inactive GDP-bound (‘off’) state and an active GTP-bound (‘on’) state [1]. This cycling is regulated by GDP/GTP exchange and GTP hydrolysis reactions, which are promoted by RAS-specific guanine nucleotide exchange factors (GEFs, such as son of sevenless, SOS) and GTPase-activating proteins (GAPs, such as neurofibromin, NF1) [2]. In its active, GTP-bound form, KRAS transduces signals to its downstream effectors, including RAF and PI3K kinases, leading to the activation of key downstream pathways such as the mitogen-activated protein kinase (MAPK) and PI3K–AKT–mTORC1 [1]. The regulation and functional output of KRAS is critically dependent on localization to the plasma membrane, which is mediated by posttranslational modification of a critical C-terminal cysteine residue and a cluster of six positively charged lysine residues. Phosphodiesterase δ (PDEδ) plays a key role in this process by binding farnesylated KRAS from the intracellular membranes and facilitating its delivery to the plasma membrane [1].

More than 25% of all solid tumors harbor mutations in the *KRAS* gene [3]. Gain-*of*-function *mutations* in *KRAS* are particularly associated with lethal forms of cancer, especially in adenocarcinoma cells of the pancreas, colon, and lung [4,5]. These mutations most frequently affect glycine 12 (G12) and glutamine 61 (Q61). Q61 is a catalytic residue essential for GTP hydrolysis; its mutation to any other amino acid effectively abolishes KRAS-mediated GTP hydrolysis [5,6]. In contrast, G12 is located in the active site between the β-/γ-phosphates of GTP, adjacent to both catalytic Q61 and the GAP arginine finger [6,7,8]. Substitution of G12, even with alanine, results in severe steric hindrance, interfering with GAP binding and impairing KRAS GTPase activity. This resistance to GAP-mediated hydrolysis [6,9] leads to the accumulation of KRAS in its active, GTP-bound state, thereby promoting persistent signaling pathways and uncontrolled tumor cell proliferation [10].

Extensive efforts to elucidate the mechanisms of intracellular trafficking, regulation, and downstream signaling of KRAS have led to the development of several therapeutic strategies [11]. The challenges of directly targeting the KRAS oncogene, along with the identification of upstream kinases such as receptor tyrosine kinases (RTKs) and downstream effectors including components of the MAPK, PI3K–AKT–mTORC1, and mTORC2–AKT pathways, have led to the development of several kinase inhibitors for targeted cancer therapy. Although KRAS is the most frequently altered oncogenic protein in solid tumors, it has long been considered ‘undruggable’ [4,12,13]. However, advances in structure-based drug design have led to the development of inhibitors selective for GDP- or GTP-bound KRAS G12 mutants [4,12,13]. Clinical trials of covalent KRAS(G12C)-specific inhibitors such as adagrasib (MRTX849) and sotorasib (AMG510) have shown promising therapeutic activity in cancers harboring this mutation. Additionally, a novel approach involves designing small molecules that ‘glue’ GTP-bound KRAS(G12D) mutants to their regulatory GAP proteins, thereby restoring GTPase activity and inhibiting oncogenic signaling [14]. This method has led to the identification of two new compounds that specifically inhibit the growth of PANC-1 cells harboring the KRAS(G12D) mutation, demonstrating significantly lower IC_50_ values and higher specificity compared to existing inhibitors like MRTX1133. However, various mechanisms contribute to acquired resistance and on-target toxicities associated with small-molecule inhibitors targeting components of the RTK–KRAS–MAPK axis [13,15,16,17]. A recent study demonstrated that both on-target and off-target mechanisms confer resistance to adagrasib [17], including secondary KRAS mutations (e.g., G12D/R/V/W, G13D, Q61H, R68S, H95D/Q/R, Y96C) and high-level amplification of the KRAS(G12C) allele. In addition, bypass resistance mechanisms, such as MET amplification, activating mutations in NRAS, BRAF, MAP2K1, and RET, oncogenic fusions involving ALK, RET, BRAF, RAF1, and FGFR3, as well as loss-of-function mutations in tumor suppressors such as NF1 and PTEN have been identified [17]. These findings underscore the urgent need for new combinatorial therapeutic strategies to prevent or overcome resistance in KRAS-mutant cancers.

Emerging evidence suggests that core or constituent signaling components assemble into macromolecular complexes and cooperate in spatially defined clusters within the cell [18]. It is therefore important to note that the stoichiometric imbalance within such complexes, whether due to gene overexpression, depletion, knockout, or targeted protein degradation, can disrupt their equilibrium and impair protein function or the activity of the entire protein complex [19]. The strength, efficiency, specificity, and fidelity of signal transduction are governed by mechanisms that enhance molecular connectivity, increase local concentration, and reduce dimensionality. One such mechanism is liquidliquid phase separation (LLPS), in which two liquid phases with distinct protein compositions emerge from a single homogeneous solution [20]. A large number of proteins, collectively referred to as accessory proteins (Supplementary Box S1), meet the criteria to promote LLPS and have been reported to act as adaptor, anchoring, docking, or scaffold proteins across diverse signaling networks [14,18,21,22]. Many of these accessory proteins orchestrate the assembly and spatiotemporal localization of key components of the RTK–KRAS–MAPK pathway [18]. Accessory proteins can be categorized into four distinct functional groups: (1) anchoring proteins, which bind to the membrane and other effectors (mostly kinases); (2) docking proteins, which interact with receptors such as RTKs or GPCRs and multiple effectors; (3) adaptor proteins, which link two signaling components (e.g., RTKs and SOS1/2); and (4) scaffold proteins, which simultaneously bind multiple partners and serve as organizing platforms for signaling complexes [18].

Although dysregulated core components of the RTK–RAS–MAPK pathway are among the most intensively studied targets for disease treatment, accessory proteins deserve greater attention. Despite substantial advances in our understanding of this signaling network, the functional significance of accessory modulators in both normal physiology and human disease, particularly cancer, remains incompletely understood. Given the critical contribution of accessory proteins to signaling fidelity and network assembly, and their operation largely from the periphery of canonical pathways, we propose that functional perturbation at specific sites within accessory proteins may ‘attenuate’ rather than completely ‘inhibit’ signaling through the hyperactivated RTK–RAS–MAPK axis [18].

Direct targeting of constituent members of the RTK–RAS–MAPK axis for disease treatment, such as in cancer, remains a major challenge. Therapies for KRAS-mutant cancers are still a significant unmet clinical need, despite the development of allele-specific inhibitors that trap and inactivate KRAS(G12C) [4,23]. Three decades of research have led to important advances in tumor treatment [24]. However, adverse side effects remain substantial, and more specific therapies could significantly reduce patient burden. Unfortunately, many of the expectations for RAS pathway-targeted drugs have not been fulfilled. High toxicity and rapid resistance acquisition have limited the success of many of the drugs developed to date [24,25]. Selective inhibition of certain accessory proteins, including CNK1, IQGAP1, KSR, and SHP2, has recently been shown to attenuate hyperactive RTK–RAS–MAPK signaling pathways in cancer cells and reduce tumor growth (Supplementary Box S2) [26,27,28,29]. For example, combining SHP2 inhibitors with MEK inhibitors has been shown to interfere with feedback reactivation by SHP2 and block the onset of resistance in KRAS-driven cancers [28,30,31,32]. Although accessory proteins are increasingly recognized as therapeutic targets in RTK–RAS–MAPK-related diseases, only a small number of accessory protein inhibitors have been identified to date [18,33].

In the present study, the effects of ablation of key accessory proteins on downstream signaling pathways, including ERK and AKT, were systematically investigated. To this end, we performed CRISPR-Cas9 knockout (KO) of *GAL3*, *PDEδ*, *NPM1*, *IQGAP1*, and *SHOC2* (Supplementary Box S1), in KRAS(G12V)-mutant adenocarcinoma cell lines. The results highlight the essential role of these accessory proteins in modulating KRAS-dependent signaling and identify them as promising candidates for combinatorial or alternative therapeutic targeting.

## 2. Materials and Methods

### 2.1. Cell Lines

The cell lines used in this study are summarized in Supplementary Table S1. All cells, including HEK-293T, Capan-1, MIA PaCa-2, PANC-1, and SW480 were obtained from the German Collection of Microorganisms and Cell Cultures in Braunschweig, Germany. SHP-77 cells were obtained from the Department of Translational Genomics at the University of Cologne in Cologne, Germany. We selected the human KRAS(G12V) mutant cancer cell lines SW480 (colon), Capan-1 (pancreas), and SHP-77 (lung) for two reasons. First, they all contain the KRAS(G12V) variant. Second, we tested various anti-KRAS antibodies and identified D2H12 (Cell Signaling, Danvers, MA, USA) as one that exhibits high selectivity against KRAS(G12V), but not other KRAS variants, such as G12D and G12C (Supplementary Figure S1). This antibody is ideal for studying KRAS(G12V) complex formation with accessory proteins.

The cells were cultured in Dulbecco’s modified Eagle medium (DMEM) supplemented with 10% fetal bovine serum (FBS) and 1% penicillin/streptomycin, except for Capan-1 and SHP-77, which were cultured in Iscove’s Modified Dulbecco’s Medium (IMDM) and Gibco Roswell Park Memorial Institute (RPMI) 1640 medium, respectively. Cells were grown in an exponential growth phase at 37 °C, 5% CO_2_, and 95% humidity.

### 2.2. CRISPR-Cas9-Mediated Gene Editing

The CRISPR-Cas9 KO was performed as previously described [34]. Briefly, purified TrueCut^TM^ Cas9 protein v2 (Thermo Fisher Scientific, Waltham, MA, USA) was incubated with TrueGuide^TM^ synthetic single guide RNA (sgRNAs) targeting the genes of the respective accessory proteins (see Supplementary Table S2) in nucleofection solution (LONZA) for 30 min at room temperature. One million cells were resuspended in the mixture and nucleofected using the 4D Nucleofector X-Unit (LONZA, Basel, Switzerland) with pulse code DN-100 for SW-480 and SHP-77, CM-130 for HEK-293, and CA-163 for CAPAN1. Cells were expanded for one week, and gene knockouts were verified by immunoblotting. The respective CRISPR-Cas9 KO was validated using immunoblotting and, in part, confocal imaging.

### 2.3. Immunoblotting and Antibodies

Immunoblotting was performed as previously described [35]. The antibodies used are listed in Supplementary Table S3. Cell lysates were prepared in lysis buffer containing 50 mM Tris-HCl, pH 7.5, 100 mM NaCl, 2 mM MgCl_2_, 1% Igepal CA-630, 10% glycerol, 20 mM beta-glycerophosphate, 1 mM sodium orthovanadate, and EDTA-free protease inhibitor cocktail (Roche, Basel, Switzerland). Cells were incubated in lysis buffer on ice for 5 min, then centrifuged at 20,000 rpm for 5 min at 4 °C to obtain the soluble protein fraction. Protein concentrations were determined by the Bradford assay (Bio-Rad, Hercules, CA, USA).

### 2.4. Cell Proliferation and Viability Assays

Proliferation of KO and wild-type (WT) cells was assessed using different protocols depending on the accessory protein and cell type. For GAL3, 1 × 10^5^ SW480 cells, and for PDEδ, 1 × 10^4^ cells were seeded per well in a 12-well plate. Proliferation was monitored for eight days. Cell numbers were estimated using both an automated counter (Bio-Rad TC20 Automated Cell Counter) and a Neubauer chamber. Trypan blue staining (1:1 ratio with the cell suspension) was used to distinguish between viable and non-viable cells. For NPM1, proliferation was analyzed in HEK-293, SHP-77, and Capan-1 cells by seeding 1 × 10^5^ cells per well in 12-well plates. Cell counts were performed on days 0, 1, 3, and 5 using a Neubauer chamber after staining with trypan blue (1:1). For IQGAP1, 5000 Capan-1 cells were seeded per well in a 96-well plate. After 6 h, 20 µL of CellTiter-Blue reagent (Promega, Madison, WI, USA) was added. Fluorescence intensity at 590 nm was measured using a Tecan Infinite M200 PRO reader (TECAN, Männedorf, Switzerland) at 0 h and again after 2 h of incubation. This procedure was repeated on days 1, 2, 3, 4, and 7. The change in fluorescence was used as a measure of cell viability. All experiments were performed using three independent biological replicates (n = 3), where each replicate represents a separate cell culture experiment conducted on different days.

### 2.5. Confocal Imaging

Immunostaining was performed as follows: The cells were washed twice with ice-cold PBS containing magnesium and calcium, then fixed with 4% formaldehyde (Merck, Darmstadt, Germany) for 20 min at room temperature. To permeabilize the cell membranes, the cells were incubated in 0.25% Triton X-100/PBS for 5 min. The cells were blocked with 3% bovine serum albumin (BSA; Merck) in PBS containing 0.25% Triton X-100 for 1 h at room temperature. The cells were then incubated with primary antibodies overnight at 4 °C, followed by staining at room temperature for 2 h. The cells were washed three times for 10 min with PBS, then incubated with secondary antibodies for 2 h at room temperature. The slides were washed three times, and ProLong^®^ Gold antifade mountant containing 4’,6-diamidino-2-phenylindole (DAPI) (Life Technologies, Waltham, MA, USA) was applied to mount the coverslips. Confocal images were obtained using an LSM 510-Meta microscope (Zeiss, Jena, Germany).

### 2.6. Cell Migration (Wound Healing or Scratch Assay)

To assess migration, 1.5 × 10^5^ cells were seeded in 12-well plates and grown to confluence. A standardized cross-shaped scratch was made using a 200 µL pipette tip. Images were captured at 0, 3, 6, 9, and 24 h using a light microscope. The width of the scratch was measured and normalized to the initial time point. Closure rates were analyzed to quantify the migration rate. Migration assays were performed in three independent biological replicates (n = 3).

### 2.7. Statistical Analysis

All experiments reported in this study were performed using three independent biological replicates (n = 3), meaning three separate cell culture experiments conducted on different days. All statistical analyses were conducted using standard methods appropriate for experimental design. Graphs for the NPM1 and IQGAP1 immunoblots were generated using Microsoft Excel. An unpaired, two-sided Student’s t-test was performed for comparisons between two groups, e.g., wild-type (WT) vs. knockout (KO). Statistical significance was indicated as follows: *p* ≤ 0.05 (*), *p* ≤ 0.01 (**), *p* ≤ 0.001 (***), and *p* ≤ 0.0001 (****). Proliferation graphs for NPM1, GAL3, and PDEδ were created using OriginPro v.2025b (OriginLab, Northampton, MA, USA). A two-way repeated measures ANOVA followed by Tukey’s post hoc test was conducted [36], with significance levels denoted as above. Immunoblot graphs for GAL3, PDEδ, and SHOC2 were prepared in OriginPro v.2025b as well. One-way ANOVA was used for group comparisons, and significance was indicated using the same star notation. For IQGAP1, in addition to immunoblots, cell viability and migration data were plotted in Microsoft Excel. We assessed statistical significance for these assays using GraphPad Prism 10. All final figures in this paper were compiled and edited using CorelDRAW, version 2020.

## 3. Results and Discussion

### 3.1. Rationale for Selecting Human KRAS(G12V)-Mutant Cancer Cell Lines

KRAS mutations are among the most common genetic alterations found in cancer, particularly in pancreatic (86%), colorectal (41%), and lung (32%) adenocarcinomas. Most of these mutations affect codon 12, including G12D (45%) and G12V (35%) in pancreatic and colorectal cancer, and G12C (46%) and G12V (23%) in lung cancer [4]. Our study initially started with the pancreatic adenocarcinoma cell line PANC-1, which harbors a homozygous KRAS(G12D) mutation and is widely used as a well-characterized in vitro model for investigating cancer mechanisms and potential therapeutic strategies (Supplementary Table S1).

Before knocking out the accessory proteins in PANC-1 cells, we visualized the endogenous KRAS protein. To this end, we tested the specificity of several polyclonal and monoclonal anti-RAS antibodies, including those previously evaluated by Der and coworkers [37]. These antibodies were tested using purified RAS family proteins expressed in *Escherichia coli*, overexpressed proteins in HEK-293 cells, and PANC-1 cell lysates (Supplementary Table S1). Two antibodies (#23-4.2 from Millipore and #C-19 from Santa Cruz), reported to be KRAS-specific [37], showed no reactivity with lysates from PANC-1 or other KRAS-mutant cell lines. To resolve this issue, we screened additional antibodies and identified one (#D2H12, Cell Signaling) that revealed high selectivity for KRAS(G12V), but not for other variants such as G12D and G12C or wild-type KRAS in HEK-293 cells (Supplementary Figure S1).

Based on these findings, we selected three KRAS(G12V)-mutant adenocarcinoma cell lines: Capan-1 (pancreatic), SW-480 (colorectal), and SHP-77 (lung) to knock out the KRAS-associated accessory proteins GAL3, PDEδ, NPM1, IQGAP1, and SHOC2, which are differentially expressed (Supplementary Figure S1). Using this model, we examined cell proliferation, migration, and downstream signaling via the MAPK, PI3K–PDK1–AKT, and mTORC2–AKT signaling pathways.

### 3.2. Galectin-3 KO Disrupts MAPK and mTORC2–AKT Signaling

**Figure 1:**
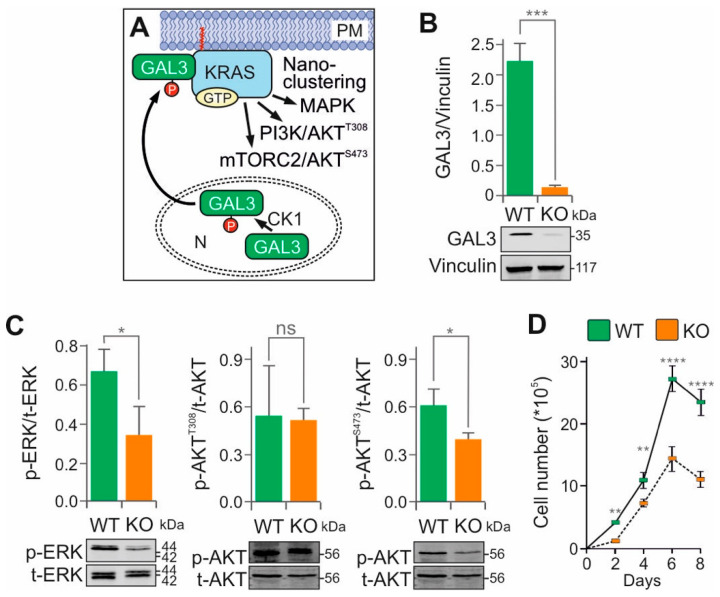
The essential modulatory function of galectin-3 in KRAS signaling in cancer cells. (**A**) Phosphorylation of GAL3 by casein kinase-1 (CK1) in the nucleus (N) induces its translocation to the plasma membrane (PM), where it is recruited into KRAS nanoclusters. (**B**) The *LGALS3* gene, encoding GAL3, was efficiently knocked out (KO) in KRAS(G12V) SW-480 cells using the CRISPR-Cas9 method. Statistical significance was determined by a one-way ANOVA (*** *p* < 0.001). (**C**) Representative immunoblots of phosphorylated ERK (p-ERK) versus total ERK (t-ERK), and phosphorylated AKT (p-AKT) versus total AKT (t-AKT), using lysates from SW-480 WT and *GAL3* KO cells. Bar graphs represent the mean of three independent experiments (n = 3), normalized to the loading control vinculin. Statistical significance was determined by a one-way ANOVA (* *p* < 0.05; ns, not significant). (**D**) Growth curves showing the mean values of daily cell counts (N = 3; Neubauer chamber) for SW-480 WT and GAL3 KO cells. Statistical analysis was performed by two-way ANOVA followed by Tukey’s post hoc test (**, *p* < 0.01, **** *p* < 0.0001). Original blots are shown in Supplementary Figure S2.

Galectin-3 (GAL3; also known as lectin L-29 or MAC-2) is a 35 kDa protein consisting of 250 amino acids that has been implicated in tumor progression and metastasis through its role in cell–cell adhesion, cell–matrix interactions, growth regulation, apoptosis, angiogenesis, and mRNA splicing [38]. Structurally, GAL3 contains an intrinsically disordered N-terminal domain and a canonical carbohydrate recognition domain (CRD) at the C-terminus, which distinguishes it from other galectin family members. The CRD forms a hydrophobic pocket proposed to accommodate the farnesyl group of KRAS [39]. Phosphorylation by casein kinase-1 (CK1) promotes GAL3 translocation from the nucleus to the plasma membrane, where it stabilizes KRAS•GTP nanoclustering (Figure 1A) [40]. It has been reported that GAL3 promotes KRAS activation but attenuates ERK but not PI3K activity [41].

We investigated the modulatory function of GAL3 in SW-480 cells by CRISPR-Cas9-mediated KO (Figure 1B). As shown in Figure 1C, *GAL3* KO significantly impaired both the MAPK and mTORC2–AKT pathways, while the PI3K–AKT pathway remained largely unaffected. Reduced proliferation of *GAL3* KO cells (Figure 1D) correlated with decreased p-ERK1/2 levels, supporting the role of GAL3 in stabilizing KRAS membrane association and downstream signaling [42]. These findings are consistent with previous studies in *GAL3* KO mouse embryonic fibroblasts, which also showed reduced p-ERK1/2 signaling [40]. An interesting finding suggests that GAL3 interacts with αvβ3 integrin, promoting KRAS-mediated AKT activation [43]. This interaction has been shown to influence resistance to EGFR inhibitors by inducing KRAS clustering in non-adherent cells [44].

Several proteins, including PDEδ, prenylin, and calmodulin, have been proposed to affect the membrane localization and intracellular trafficking of farnesylated KRAS [45]. In contrast to these factors, which enhance KRAS dissociation from the plasma membrane, GAL3 specifically reduces the dissociation rate of activated KRAS. A dominant negative mutant of GAL3 GAL3(V125A) has been shown to bind GTP-bound KRAS, impair nanocluster formation and MAPK activity, and suppress cell growth [39]. However, in our study, GST pulldown assays using bacterially expressed and purified GAL3 and KRAS proteins failed to demonstrate a direct interaction with either GDP- or GTP-bound KRAS, possibly due to the absence of post-translational modifications.

KRAS nanoclusters refer to nanoscale aggregates of KRAS on the plasma membrane that facilitate the assembly of signaling complexes [46]. Several studies have implicated GAL3 in the stabilization of these nanoclusters (Figure 1A). It is proposed that GTP-bound KRAS recruits GAL3 from the cytosol to the membrane, where GAL3 becomes a component of the nanocluster, thereby modulating the magnitude of KRAS•GTP signaling output in a concentration-dependent manner [39,47,48]. Our data are consistent with this proposed scaffolding role, although the precise molecular mechanisms underlying GAL3-mediated nanocluster formation remain to be elucidated.

### 3.3. PDE6D (PDEδ) KO Impairs MAPK and AKT Signaling

**Figure 2:**
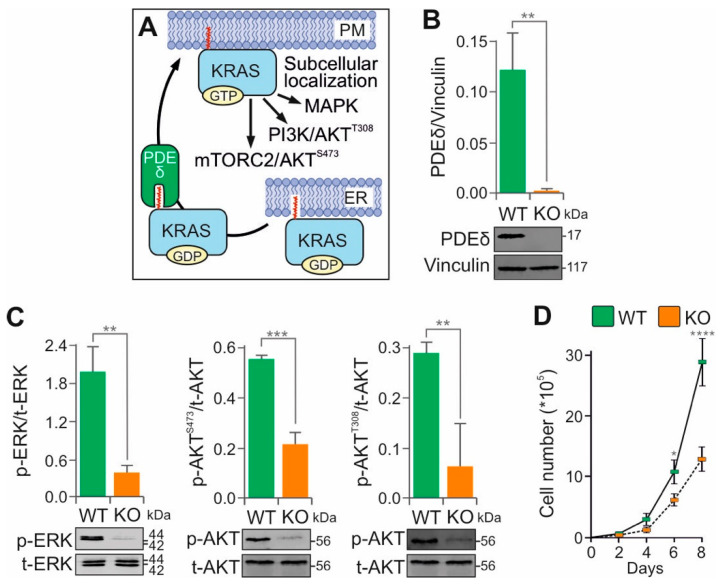
PDEδ is essential for KRAS(G12V) signaling in SW-480 cancer cells. (**A**) Schematic illustrating the proposed role of PDEδ in regulating KRAS spatial dynamics and membrane association. Statistical significance was assessed by a one-way ANOVA (** *p* < 0.01). (**B**) The *PDE6D* gene, encoding PDEδ, was efficiently knocked out (KO) in KRAS(G12V) SW-480 cells using CRISPR-Cas9. (**C**) Bar graphs represent the mean of three independent experiments (n = 3), normalized to the loading control vinculin. Statistical significance was assessed by a one-way ANOVA (** *p* < 0.01, *** *p* < 0.001). (**D**) Growth curves show the mean daily cell counts (N = 3; Neubauer chamber) for SW-480 WT and *PDEδ* KO cells. Statistical analysis was performed by two-way ANOVA followed by Tukey’s post hoc test (* *p* < 0.05, **** *p* < 0.0001). Original blots are shown in Supplementary Figure S3.

Phosphodiesterases (PDEs) comprise a large family of enzymes with 11 isoenzyme classes and over 50 subunits that play essential roles in various signaling pathways [49]. Among them, PDEδ (also known as PDE6D or PrBP) is particularly important for the spatial organization of prenylated KRAS by facilitating its cytoplasmic diffusion and regulating its dynamic association with cellular membranes (Figure 2A) [50].

To investigate the role of PDEδ in KRAS-driven signaling, we generated PDE6D KO cells using CRISPR-Cas9 technology in the KRAS(G12V)-mutant SW-480 colorectal adenocarcinoma cell line (Figure 2B). Immunoblotting revealed a significant reduction in phosphorylated ERK and AKT levels upon PDE6D knockout, indicating impaired MAPK and PI3K–AKT signaling (Figure 2C). This signaling impairment correlated with a substantial decrease in cell proliferation (Figure 2D), suggesting a critical role for PDEδ in maintaining KRAS effector pathways.

Our results are consistent with previous studies showing that impairment of the PDE6D–KRAS interaction attenuates KRAS-driven signaling [50]. However, they contrast with recent siRNA-mediated knockdown experiments that showed minimal effects on ERK activity [51]. This discrepancy may be due to the incomplete suppression achieved by transient knockdown, leaving residual PDEδ activity sufficient to support partial KRAS membrane localization. In addition, variation in dependence on PDEδ between different KRAS-mutant cell types may also contribute to these divergent observations.

The coordinated reduction in p-AKT(Ser473) and p-AKT(Thr308) suggests that PDEδ regulates AKT signaling at multiple levels, including upstream PI3K–PDK1 and mTORC2 inputs. Given that small-molecule inhibitors of PDEδ have been shown to impair KRAS membrane localization and reduce tumor growth in xenograft models [50]. This supports PDEδ as a promising therapeutic target in KRAS-mutant cancers, particularly colorectal adenocarcinoma.

### 3.4. Nucleophosmin KO Affects RAS Signaling in HEK-293 Cells, but Not in KRAS-Mutant Cancer Cells

**Figure 3:**
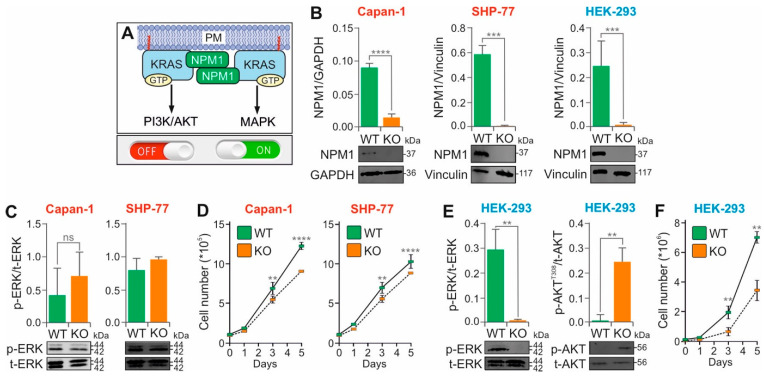
Reciprocal modulation of MAPK and PI3K-AKT signaling pathways by NPM1 in HEK-293 cells. (**A**) Schematic representation of the proposed role of NPM1 in stabilizing KRAS at the plasma membrane (PM), promoting activation of the MAPK pathway while inhibiting PI3K–AKT signaling. (**B**) Confirmation of *NPM1* KO in KRAS(G12V)-mutant cell lines (Capan-1 and SHP-77) and KRAS wild-type HEK-293 cells using CRISPR-Cas9 technology. NPM1 KO was verified by immunoblotting. (**C**) Representative immunoblots of phosphorylated ERK (p-ERK) versus total ERK (t-ERK), using lysates from Capan-1 (n = 3), SHP-77 (n = 2) and NPM1 (n = 3). (**D**) Growth curves showing the mean values of daily cell counts (N = 3; Neubauer chamber) for Capan-1 and SHP-77 WT and NPM1 KO cells. (**E**) Representative immunoblots of p-ERK versus t-ERK and phosphorylated AKT (p-AKT) versus total AKT (t-AKT), using lysates from HEK-293 WT and NPM1 KO cells. (**F**) Growth curves showing the mean values of daily cell counts (N = 3; Neubauer chamber) for HEK-293 WT and NPM1 KO cells. Bar graphs represent the mean of three independent experiments (n = 3), normalized to the loading control GAPDH or vinculin. For growth curves, statistical analysis was performed using two-way ANOVA followed by Tukey’s post hoc test (** *p* < 0.01, *** *p* < 0.001, **** *p* < 0.0001, ns, not significant). Original blots are shown in Supplementary Figure S4.

Nucleophosmin (NPM1; also known as B23, numatrin, or NO38) is a 37 kDa multifunctional phosphoprotein of 294 amino acids. It is predominantly localized to the nucleolus and plays critical roles in RNA processing events, including transcription, ribosome biogenesis, mRNA stability, translation, and miRNA regulation [52]. Notably, the KRAS-responsive lncRNA KIMAT1, a MYC target, promotes lung tumorigenesis by enhancing the processing of oncogenic miRNAs through the stabilization of NPM1 [53,54]. Although NPM1 functions primarily in the nucleus, it shuttles between the nuclear and cytoplasmic compartments and can translocate to the plasma membrane. Mutant forms of NPM1 have been shown to enhance RAS–MAPK signaling, thereby promoting adhesion, migration, and invasion in acute myeloid leukemia (AML) cells [55]. Furthermore, genetic ablation of *NPM1* reduces tumor progression in non-small cell lung cancer (NSCLC), validating NPM1 as a therapeutic target in KRAS-driven tumors [56]. Interestingly, NPM1 has been reported to stabilize KRAS association with the cytoplasmic leaflet of the plasma membrane, thereby modulating MAPK signaling (Figure 3A) [48].

To investigate the functional role of NPM1 in KRAS signaling, we knocked out NPM1 in Capan-1, SHP-77, and HEK-293 cell lines using CRISPR-Cas9 (Figure 3B). The KO efficiency was confirmed by immunoblotting. In the KRAS(G12V)-mutant adenocarcinoma cell lines Capan-1 and SHP-77, NPM1 KO had no significant effect on MAPK signaling as measured by p-ERK1/2 levels (Figure 3C), although a modest but reproducible reduction in proliferation was observed (Figure 3D). In contrast, *NPM1* KO in the KRAS wild-type HEK-293 cell line resulted in a significant reduction in p-ERK1/2 and a striking increase in p-AKT (Figure 3E), accompanied by a substantial decline in proliferation (Figure 3F). These results suggest that *NPM1* KO does not reverse the strong activation of signaling in KRAS-mutant cancer cells but instead alters the downstream signaling in KRAS wild-type HEK-293 cells. In this context, NPM1 appears to promote the RAS–MAPK pathway while suppressing the RAS–PI3K–AKT pathway (Figure 3A).

The role of NPM1 in cancer, particularly its mutations and interactions with KRAS, has received considerable attention due to its impact on signaling pathways involved in cell proliferation and differentiation. In this study, no significant changes in ERK phosphorylation were observed upon *NPM1* KO in the KRAS(G12V)-mutant adenocarcinoma cell lines. Previous studies have reported NPM1 overexpression in various malignancies [57] and identified frameshift mutations in approximately 35% of adult AML cases [58,59]. These mutations often co-occur with activating KRAS mutations [60,61], suggesting a potential cooperative role in leukemogenesis. Genetic ablation of *NPM1* in a mouse model of KRAS-mutant adenocarcinoma has been shown to shift cancer cell metabolism from aerobic glycolysis to oxidative phosphorylation and reduce tumor proliferation [56], supporting its candidacy as a potential therapeutic target in KRAS-driven cancers. Although ectopic NPM1 expression has been reported to increase p-ERK levels [48] we did not observe changes in ERK phosphorylation in the KRAS(G12V) background, suggesting that constitutively active KRAS maintains MAPK pathway activation independent of NPM1 status.

In contrast, *NPM1* KO in the KRAS wild-type HEK-293 cell line significantly altered downstream signaling, with decreased ERK phosphorylation and increased AKT phosphorylation. This finding supports the idea that NPM1 is required to stabilize KRAS at the plasma membrane [62]. Immunoprecipitation studies have shown that an AML-associated NPM1 mutant (a four-nucleotide insertion in exon 11) interacts with KRAS [63]. Overexpression of this mutant increases p-ERK levels [55], which is consistent with our findings. It has also been suggested that this mutant promotes AML cell invasiveness by upregulating matrix metalloproteinases (MMPs) through activation of the RAS–MAPK pathway, further highlighting its role in leukemogenesis [55]. However, our GST pulldown assays with purified proteins did not confirm a direct interaction between wild-type NPM1 and KRAS.

Notably, *NPM1* KO in HEK-293 cells also resulted in increased AKT phosphorylation, likely due to the destabilization of PTEN, a key negative regulator of the PI3K pathway. NPM1 has been reported to interact with PTEN and regulate its stability via ubiquitination [64]. In the absence of NPM1, PTEN degradation may result in enhanced PI3K–AKT signaling.

In conclusion, NPM1 appears to modulate both the MAPK and PI3K-AKT pathways in a KRAS context-dependent manner. In KRAS(G12V)-mutant cell lines, its KO has minimal effect due to constitutive downstream, whereas in KRAS wild-type cells, NPM1 promotes MAPK activation and restrains AKT signaling. These findings support a broader role for NPM1 in KRAS signaling modulation and warrant further investigation of its mechanism and therapeutic potential.

### 3.5. IQGAP1 KO Does Not Alter MAPK Signaling, but Positively Affects PI3K-PDK1-AKT Activation

Activation of RAF kinase at the plasma membrane through direct interaction with KRAS•GTP is a well-established mechanism [65]. Upon activation, BRAF/RAF1 heterodimers phosphorylate MEK1/2, which then phosphorylate ERK1/2 at the TEY motif. Activated ERK1/2 are then distributed to various subcellular compartments, where they phosphorylate downstream substrates. The assembly of macromolecular complexes involving MAPK components and their interaction with RAS nanoclusters at the membrane defines the RAS–MAPK signaling axis. This process is supported by homo- and heterodimerization of the signaling proteins and orchestrated by accessory proteins that provide spatial and temporal precision, as well as signal fidelity and amplification [18]. One of the most prominent scaffold proteins involved in this regulation is IQ Motif Containing GTPase Activating Protein 1 (IQGAP1), also known as p195 or SAR1, a multidomain protein composed of 1657 amino acids with a molecular weight of approximately 180 kDa [18,66,67].

**Figure 4:**
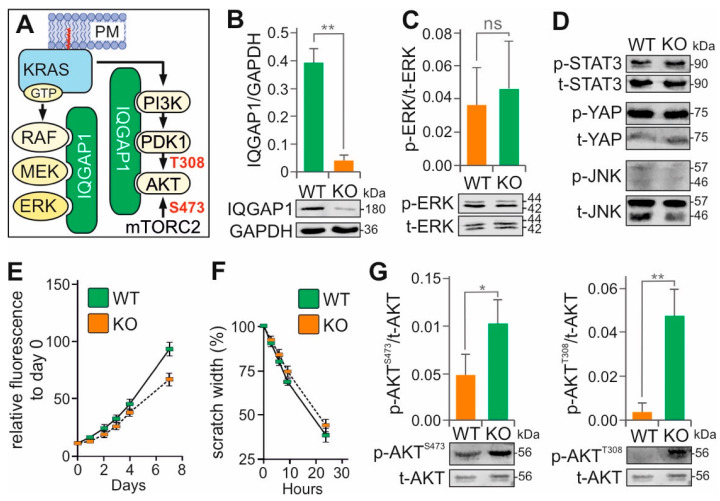
The regulatory role of IQGAP1 in PI3K–AKT signaling in KRAS(G12V)-mutant Capan-1 cells. (**A**) IQGAP1 is proposed to function as a scaffold protein for components of both the MAPK (RAF, MEK, ERK) and PI3K–AKT (PI3K, PDK1, AKT) pathways. (**B**) The *IQGAP1* gene was efficiently knocked out (KO) in KRAS(G12V) Capan-1 cells using the CRISPR-Cas9 method. (**C**) Representative immunoblots of phosphorylated ERK (p-ERK) versus total ERK (t-ERK), using lysates from Capan-1 WT and *IQGAP1* KO cells. (**D**) Representative immunoblots phosphorylated STAT3 (p-STAT3), YAP (p-YAP), and JNK (p-JNK) versus total STAT3 (t-STAT3), YAP (t-YAP), and JNK (t-JNK), using lysates from Capan-1 WT and *IQGAP1* KO cells. (**E**) Cell viability was measured for Capan-1 WT and *IQGAP1* KO cells using the CellTiter-Blue assay (n = 3). Fluorescence was normalized to day 0 to reflect the relative increase in viable cells over time. (**F**) Cell migration was assessed using a scratch assay. The plots show the mean values of wound closure (n = 3) over time for Capan-1 WT and *IQGAP1* KO cells. (**G**) Representative immunoblots of phosphorylated AKT (p-AKT) versus total AKT (t-AKT), using lysates from Capan-1 WT and *IQGAP1* KO cells. Bar graphs represent the mean of three independent experiments (n = 3), normalized to the loading control GAPDH. Statistical significance was assessed using an unpaired two-tailed *t*-test (* *p* < 0.05, ** *p* < 0.01; ns, not significant). Original blots are shown in Supplementary Figure S5.

IQGAP1 has been reported to scaffold and facilitate the RAF–MEK–ERK signaling by interacting with the epidermal growth factor receptor (EGFR) [68,69] and to coordinate PI3K signaling by assembling a multienzyme complex that promotes PIP_3_ generation and subsequent AKT activation (Figure 4A) [70]. With over one hundred documented binding partners [71], IQGAP1 is involved in cytoskeletal remodeling, cell proliferation, differentiation, and other regulatory functions that vary depending on the cell type and physiological context. Because of its central role in tumorigenesis, IQGAP1 and its associated pathways are being evaluated as potential targets for cancer therapy [72].

In this study, we generated *IQGAP1* KO in the Capan-1 cell line using CRISPR-Cas9. The *IQGAP1* KO was validated using both immunoblotting (Figure 4B) and confocal imaging (Supplementary Figure S2). Unexpectedly, immunoblot analysis revealed that *IQGAP1* KO did not affect ERK phosphorylation. (Figure 4C). Therefore, the phosphorylation status of other signaling proteins, including JNK, YAP, and STAT3, was measured, which also remained unchanged (Figure 4D). Cell proliferation and migration also showed no significant difference between *IQGAP1* KO and wild-type cells (Figure 4E,F). In contrast, we observed a significant increase in AKT phosphorylation at T308, the PDK1 target site within the PI3K pathway, in *IQGAP1* KO cells compared to wild-type controls (Figure 4G). This suggests that IQGAP1 acts as a negative modulator of the KRAS–PI3K–PDK1–AKT signaling axis in the KRAS(G12V)-mutant Capan-1 cells.

IQGAP1 is frequently overexpressed in various cancers, including pancreatic adenocarcinoma [73,74], colorectal cancer [75], hepatocellular carcinoma [76], ovarian cancer [77], and glioma [78]. As a scaffold protein for MAPK components [79,80], it was initially thought to interact with ERK via the WW domain, although recent evidence suggests that the IQ domain is both necessary and sufficient for high-affinity binding [68,80,81]. IQGAP1 also facilitates crosstalk between mTOR and AKT [82] and directly promotes PIP_3_ generation by binding to PI3K [70,83,84].

Interestingly, despite the proposed scaffolding functions of IQGAP1, we did not observe changes in ERK phosphorylation following *IQGAP1* knockout. Instead, levels of phosphorylated AKT at T308 (p-AKT^T308^) were significantly increased, with a modest increase also observed at S473 (p-AKT^S473^). These results differ from previous reports characterizing IQGAP1 as a positive regulator of AKT signaling. For example, siRNA-mediated knockdown of IQGAP1 in Capan-1 cells was reported to leave p-AKT levels unaffected while reducing p-ERK levels [85]. In KRAS wild-type cells, high levels of IQGAP1 may favor the PI3K–AKT signaling by interacting with FOXO1, a downstream target of AKT, while interfering with the scaffolding of the MAPK pathway [86]. In KRAS-mutant cells, IQGAP1 may instead promote ERK signaling while limiting AKT activation. Thus, loss of IQGAP1 could remove this bias and shift the balance in favor of PI3K–AKT pathway activation. An additional explanation may involve a feedback mechanism regulated by p70S6K. IQGAP1 has been proposed to affect p70S6K, and its loss may relieve a negative feedback loop, thereby increasing AKT activity [87].

### 3.6. SHOC2 KO Selectively Disrupts KRAS(G12V)-Driven MAPK Signaling

**Figure 5:**
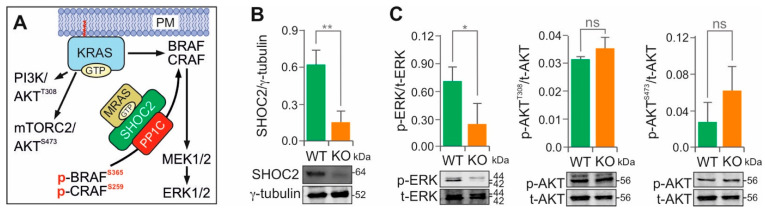
SHOC2 selectively modulates MAPK signaling in KRAS(G12V) SHP-77 cells. (**A**) Schematic representation of SHOC2-mediated RAF activation via dephosphorylation by the SHOC2–MRAS–PP1C complex. (**B**) The *SHOC2* gene was knocked out (KO) in KRAS(G12V) adenocarcinoma SHP-77 cells using the CRISPR-Cas9 method. (**C**) Representative immunoblots of phosphorylated ERK (p-ERK) versus total ERK (t-ERK), and phosphorylated AKT (p-AKT) versus total AKT (t-AKT), using lysates from SHP-77 WT and *SHOC2* KO cells. Bar graphs represent the mean of three independent experiments (n = 3), normalized to the loading control γ-tubulin. Statistical significance was assessed by a one-way ANOVA (* *p* < 0.05, ** *p* < 0.01; ns, not significant). Original blots are shown in Supplementary Figure S6.

SHOC2 (also known as SUR-8 or SOC2) is a scaffold protein that facilitates activation of the RAS–MAPK pathway by coordinating the assembly of signaling complexes. It forms a ternary complex with protein phosphatase 1 (PP1C) and GTP-bound MRAS, a member of the RAS family. This SHOC2–MRAS–PP1C complex specifically dephosphorylates inhibitory serine residues on RAF kinases, such as p-CRAF(S259) and p-BRAF(S365) (Figure 5A) [88,89,90]. This dephosphorylation step relieves autoinhibition, thereby enabling RAF activation and subsequent MEK1/2 phosphorylation. The functional importance of this complex is underscored by gain-of-function mutations in all three components in Noonan syndrome, which enhance complex formation and MAPK signaling [91,92].

After confirming SHOC2 expression in various cell lines (Supplementary Figure S1), we generated *SHOC2* KO in the KRAS(G12V) adenocarcinoma cell line SHP-77 using CRISPR-Cas9 (Figure 5B). As shown in Figure 5C, *SHOC2* KO resulted in a marked reduction in ERK phosphorylation, whereas AKT phosphorylation at both T308 and S473 remained unchanged. These results demonstrate that SHOC2 specifically modulates the MAPK axis downstream of KRAS, without affecting the AKT pathways.

Our results are consistent with previous studies demonstrating the essential role of SHOC2 in the activation of the MAPK pathway [93]. SHOC2 facilitates the recruitment of PP1C to inactive BRAF and CRAF, thereby promoting the dephosphorylation and activation of RAF kinases (Figure 5A). SHOC2 expression and mutation status vary among tumor types, and alterations in the axis are implicated in multiple cancer subtypes [94,95]. SHOC2 overexpression has been associated with increased ERK phosphorylation in several cancers, including colorectal, lung, and breast tumors [89,93,96,97,98]. Beyond cancer, SHOC2 deregulation has been implicated in developmental disorders. Germline gain-of-function mutations in *SHOC2* cause Noonan-like syndrome with loose anagen hair, a distinct RASopathy characterized by aberrant MAPK signaling [99,100,101].

## 4. Conclusions and Future Perspectives

In this study, we highlight a class of modulatory proteins, termed ‘accessory proteins’, that are increasingly recognized as promising therapeutic targets in RAS–MAPK-driven diseases. These proteins do not function as direct components of canonical signaling cascades, but instead coordinate the assembly and spatiotemporal localization of key signaling molecules, thereby shaping overall signaling output [18]. Accessory proteins can be classified into four functional subtypes, as outlined in Supplementary Box S1.

Our results demonstrate that the loss of specific KRAS-associated accessory proteins significantly alters KRAS signaling outputs in both KRAS(G12V)-mutant adenocarcinoma cells and KRAS wild-type HEK-293 cells. In particular, we identified GAL3, PDEδ, SHOC2, and IQGAP1 as key modulators of KRAS-centric interaction networks, positioning them as strong candidates for therapeutic intervention (Supplementary Box S2).

These proteins also offer opportunities to refine combination therapeutic strategies aimed at overcoming resistance to KRAS-targeted therapies. Notably, previous studies have shown that depletion of accessory proteins such as SHOC2 can reduce the likelihood of acquired drug resistance [90,102,103,104]. In addition, our findings, including the reciprocal modulation of MAPK and AKT signaling upon *NPM1* KO in KRAS wild-type cells, and the unexpected increase in p-AKT upon *IQGAP1* KO, provide novel insights. These results support the concept that certain accessory proteins may function as molecular switches that selectively activate or repress specific signaling pathways depending on the cellular context.

Although the last three decades of research have led to major advances in cancer treatment, more effective therapeutic strategies are still needed, particularly for cancers harboring KRAS mutations. Several accessory proteins have been suggested as promising targets in RAS-mutant cancers, but only a small number of inhibitors have been discovered.

A key advantage of targeting modulatory proteins rather than core or constituent pathway components is that hyperactive signaling can be attenuated to physiological levels rather than blocked completely. This strategy may reduce toxicity and limit the compensatory feedback reactivation often observed with direct kinase inhibition. For example, *KSR* KO in mice does not eliminate ERK phosphorylation but reduces RAS-driven tumorigenesis and is well tolerated during development [24,105].

One illustrative example is the scaffolding protein SHOC2. Its depletion enhances the therapeutic effect of MEK inhibitor treatment by interfering with feedback reactivation through the RAF pathway [106]. SHOC2 forms a holoenzyme complex with PP1C and MRAS that enables RAF dimerization by dephosphorylating inhibitory serine residues. While *SHOC2* KO is embryonic lethal in mice, it is tolerated in adult animals and human cell lines, and its loss inhibits the growth of RAS mutant cancer cells [106]. In addition to its role in cancer, SHOC2 mutations have been implicated in developmental disorders such as Mazzanti syndrome and prenatal-onset hypertrophic cardiomyopathy [99,101]. These mutations cause persistent membrane localization or enhanced binding of MRAS and PPP1CB, highlighting the finely tuned regulatory role of SHOC2 in RAS-MAPK signaling.

Another example is the anchoring protein CNK1, which localizes to the membrane via its pleckstrin homology (PH) domain and promotes RAF activation by binding to both RAS and RAF via its N- and C-terminal domains [107]. The PH domain inhibitor PHT-7.3 prevents CNK1 from colocalizing with membrane-localized RAS and selectively inhibits the proliferation of KRAS mutant, but not wild-type, cancer cell lines [29].

A further example is the tyrosine phosphatase SHP2, which plays a key role in integrating RTK signals and is essential for cell growth and proliferation. Blocking SHP2 in cancers driven by RTK activation has been shown to attenuate tumor growth. Allosteric inhibitors of SHP2 suppress signaling through the MAPK pathway and inhibit the proliferation of RTK-activated tumors. This offers a strategy to overcome resistance in cancer treatments [108]. It also has potential for combination therapies. SHP2 inhibition could enhance the efficacy of other treatments, such as MEK inhibitors, by reducing MAPK and mTOR signaling activities [109].

As shown in our study, PDEδ can significantly affect both pathways and could be used in cancer therapy. Moreover, the combination of inhibition of PDEδ function, when combined with sildenafil, which activates PKG2, synergistically suppresses KRAS-driven tumor growth in preclinical models [110].

Collectively, the results of this study provide a first functional map of KRAS-associated accessory proteins and their influence on MAPK and AKT signaling. By uncovering their distinct and context-dependent regulatory roles, we highlight these modulators as attractive but underexplored therapeutic targets. Future efforts to develop selective inhibitors of accessory proteins may pave the way for more effective and durable treatment strategies in KRAS-driven cancers.

## Data Availability

All data generated or analyzed during this study are included in this published article and its Supplementary Materials. Additional information is available from the corresponding author upon reasonable request.

## Supplementary Materials

The following supporting information can be downloaded at: https://www.mdpi.com/article/10.3390/cells15020190/s1, Figure S1: Relative expression of KRAS and KRAS-related accessory proteins in cancer cell lines; Figure S2. Validation of IQGAP1 Knockout Using Confocal Microscopy; Figure S3. Scratch Assay; Table S1: Human cell lines used in this study; Table S2: Guide-RNAsa used in this study; Table S3: Antibodies used in this study; Box S1. Accessory proteins related to the RTK-RAS-MAPK signaling cascade can be divided into at least four subgroups; Supplementary Box S2. Inhibitors or Compounds. References [111,112,113,114,115,116,117,118,119,120,121,122,123,124,125,126,127] are cited in supplementary materials.

## Abbreviations

The following abbreviations are used in this manuscript:

AKT	Protein kinase B
ALK	Anaplastic lymphoma kinase
AML	Acute myeloid leukemia
BRAF	B-Rapidly accelerated fibrosarcoma Serine/threonine-protein kinase
Cas9	CRISPR-associated protein 9
cGMP	Cyclic guanosine monophosphate
CK1	Casein Kinase 1
CNK1	Connector enhancer of kinase suppressor of RAS 1
CO_2_	Carbon dioxide
CRAF	C-Rapidly accelerated fibrosarcoma Serine/threonine-protein kinase
CRD	Carbohydrate recognition domain
CRISPR	Clustered regularly interspaced short palindromic repeat
DMEM	Dulbecco’s Modified Eagle Medium
EGF	Epidermal growth factor
EGFR	Epidermal growth factor receptor
ERK	Extracellular signal-regulated kinase
FBS	Fetal bovine serum
FDA	Food and Drug Administration
FGFR3	Fibroblast growth factor receptor 3
FOXO1	Forkhead box protein O1
GAL3	Galectin-3
GAP	GTPase-activating protein
GAPDH	Glyceraldehyde 3-phosphate dehydrogenase
GDP	Guanine diphosphate
GEF	Guanine nucleotide exchange factor
GPCR	G protein-coupled receptor
GRB2	Growth factor receptor-binding protein 2
GST	Glutathione S-transferase
GTP	Guanine triphosphate
HEK-293	Human embryonic kidney 293
lncRNA	long non-coding RNA
IQGAP1	IQ Motif-Containing GTPase-Activating Protein 1
JNK	c-Jun N-terminal kinase
kDa	Kilodalton
KIMAT1	KRAS-induced MYC-activated Transcript 1
KO	Knockout
KRAS	Kirsten rat sarcoma
KSR1	Kinase suppressor of RAS-1
LLPS	LiquidLiquid phase separation
MAP2K1	Mitogen-activated protein kinase kinase 1
MAPK	Mitogen-activated protein kinase
MEK	Dual specificity mitogen-activated protein kinase kinase
MET	Mesenchymal–Epithelial Transition factor
MMPs	Matrix metalloproteinases
MRAS	Muscle RAS oncogene homolog
mRNA	Messenger Ribonucleic Acid
mTOR	Mammalian target of rapamycin
mTORC2	Mammalian target of rapamycin complex 2
MYC	Avian Myelocytomatosis Viral Oncogene Homolog
NF1	Neurofibromatosis type 1
NPM1	Nucleophosmin 1
NRAS	Neuroblastoma RAS viral oncogene homolog
NSCLC	Non-small cell lung cancer
PDE	Phosphodiesterase
PDE6δ	Delta subunit of phosphodiesterase 6
PDK1	3-phosphoinositide-dependent protein kinase 1
PH	Pleckstrin homology
PI3K	Phosphatidylinositol 3-kinase
PIP3	Phosphatidylinositol (3,4,5)-triphosphate
PP1C	Protein phosphatase 1 catalytic subunit
PP1CB	Protein phosphatase 1 catalytic subunit beta
PTEN	Phosphatase and tensin homolog
RAF	Rapidly accelerated fibrosarcoma
RAS	Rat sarcoma
RET	Rearranged during transfection (proto-oncogene tyrosine-protein kinase receptor)
RNA	Ribonucleic Acid
RTK	Receptor tyrosine kinase
sgRNA	Single-guide RNA
SHP-77	Shadyside Hospital, Pittsburgh 77
SHP2	SH2 domain-containing tyrosine phosphatase 2
siRNA	Small Interfering RNA
SOS	Son of sevenless
SOS1/2	Son of sevenless homolog 1/2
STAT3	Signal Transducer and Activator of Transcription 3
WT	Wild-Type
YAP	Yes1-associated transcriptional regulator
