# Supplementary material for: Unraveling the Impact of KRAS Accessory Proteins on Oncogenic Signaling Pathways
Source: Cells. 2026 Jan 20;15(2):190. doi: 10.3390/cells15020190 (PMC12839328; doi:10.3390/cells15020190)
Supplement: Supplementary file 1 [file cells-15-00190-s001.zip › cells-4043564-supplementary.pdf]

Supplementary Materials

Table S1. Human cell lines used in this study.

Cell line[a]	Accession number	Origin/Tissue	KRAS mutation[b]	Other mutations[b]
HEK-293T	CVCL_0063	Kidney	WT	SV40 large T antigen
PANC-1	CVCL_0480	Pancreas	G12D (1)	CDKN2a (2), TP53 (2)
MIA PaCa-2	CVCL_0428	Pancreas	G12C (2)	CDKN2a (2), TP53 (2)
SHP-77	CVCL_1693	Lung	G12V (2)	ABL1 (2), RAC1 Y32C (2), TP53 (2)
SW480	CVCL_0546	Colon	G12V (2)	APC (1), TP53 (2)
Capan-1	CVCL_0237	Pancreas	G12V (2)	BRCA2 (2), SMAD4 (2), TP53 (2)

[a] Resource: Cellosaurus. [b] Numbers in brackets represent the number of mutant alleles (1, heterozygous; 2, homozygous); amp., amplification; HEK, Human embryonic kidney 293T.

Table S2. Guide-RNAs[a] used in this study.

PDEδ (PDE6D)	ACCTTCGGGATGCTGAGACA
GAL3 (LGALS3)	CATGATGCGTTATCTGGGTC
IQGAP1	GGGGTCTACCTTGCCAAACT
NPM1	TGCAGAGTCAGAGATGAAG
SHOC2	GGAAGAGAATTCAATGCGTT

[a] Guide RNAs have been ordered from Thermofisher Scientific. (https://www.thermofisher.com/de/de/home/life-science/genome-editing/crispr-libraries/trueguide-grnas.html)

Table S3. Antibodies used in this study.

Antibody[a]	Species	Supplier	Reference
AKT	Mouse	Cell Signaling	2920S
pAKT (T308)	Rabbit	Cell Signaling	2965S
pAKT (S473)	Rabbit	Cell Signaling	9271, 4060S
ERK1/2	Rabbit	Cell Signaling	9102
pERK1/2	Mouse	Cell Signaling	9106S
Galectin-3	Mouse	Abcam	ab2785
GAPDH	Rabbit	Cell Signaling	2118S
GAPDH	Mouse	Thermo Fisher Scientific	39-8600
IQGAP1	Mouse	Abcam	ab56529
IQGAP1	Rabbit	Novus Biologicals	NBP1-06529
JNK	Rabbit	Cell Signaling	9252S
pJNK (T183/Y185)	Rabbit	Cell Signaling	9251
$KRAS^{G12V}$	Rabbit	Cell Signaling	D2H12
NPM1	Mouse	Abcam	ab10530
Pan-RAS	Mouse	Millipore	05-516
Paxillin	Mouse	Sigma-Aldrich	MAB3060
PDEδ	Rabbit	Abcam	ab5665
SHOC2	Rabbit	Sigma-Aldrich	HPA009164
STAT3	Mouse	Cell Signaling	9139
pSTAT3	Rabbit	Cell Signaling	4324
α-tubulin	Rabbit	Abcam	ab52866
γ-tubulin	Mouse	Sigma-Aldrich	T5326
Vinculin	Mouse	Sigma-Aldrich	V9131
YAP	Rabbit	Cell Signaling	4912
pYAP (S127)	Rabbit	Cell Signaling	4911
Alexa Fluor 546	-	Thermo Fisher Scientific	A22283
DAPI	-	Thermo Fisher Scientific	D1306
IR©Dye 680RD anti-mouse	Donkey	Licor	926-68072
IR©Dye 800CW anti-rabbit	Donkey	Licor	926-32213

[a] The anbibody diultions were 1:1000, except for IR©Dye 680RD anti-mouse and IR©Dye 800CW anti-rabbit, which were 1:10.000.

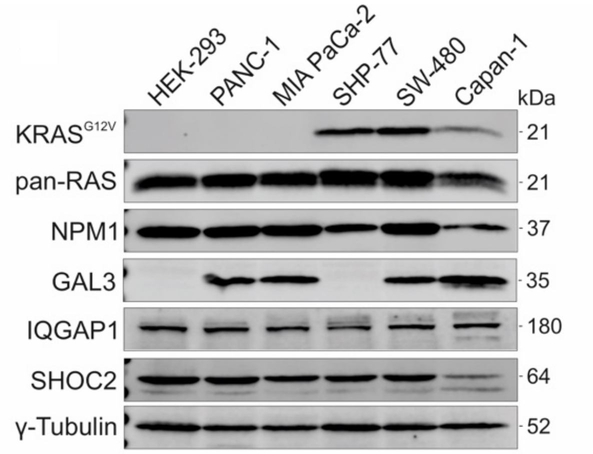

Figure S1. Relative expression of KRAS and KRAS-related accessory proteins in cancer cell lines. (A) Immunoblotting was performed different antibodies as indicated and listed in Supplementary Table S3. Total cell lysates were adjusted to a 50 mg/ml protein concentration.

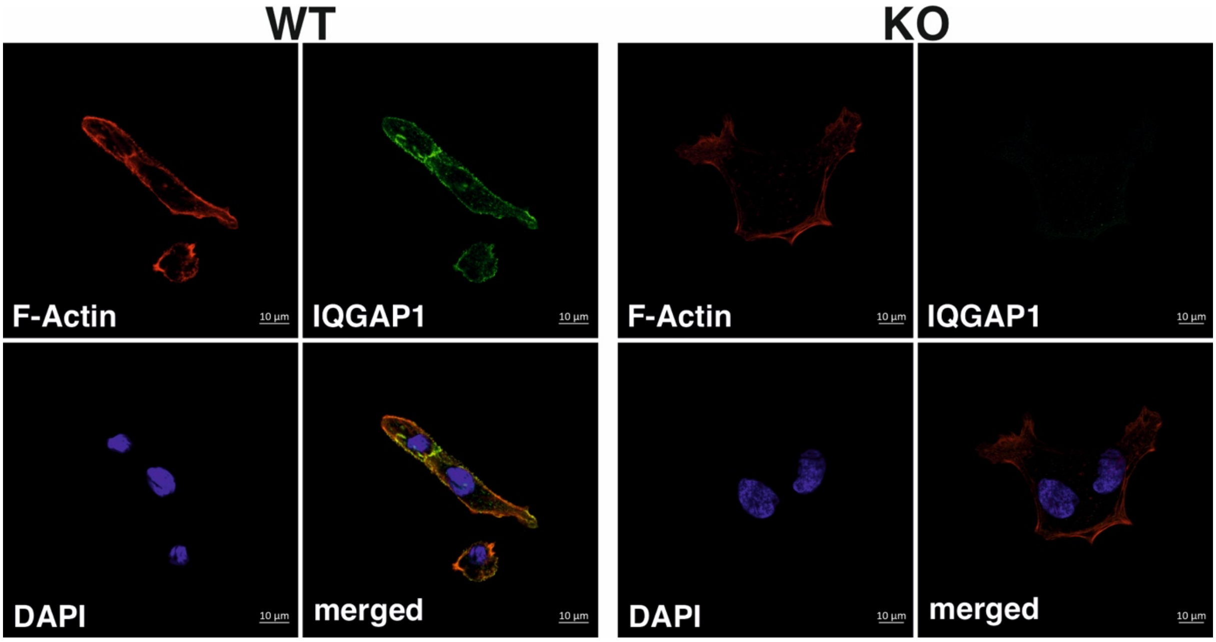

Figure S2. Validation of IQGAP1 Knockout Using Confocal Microscopy. IQGAP1 (green; anti-IQGAP1: #NBP1-06529, Novus Biologicals) actin (red; Alexa Fluor 546: #A22283, Thermo Fisher Scientific), and DNA (blue; DAPI: #D1306, Thermo Fisher Scientific) were stained in wild-type (WT) and knockout (KO) Capan-1 cells using DAPI (blue). The same color channel settings were used in the Zen 3.4 Blue Edition program. One representative cell was selected for each WT and KO cell. The color channels were displayed individually and merged to create a multichannel image. Scale bar: 10 μm.

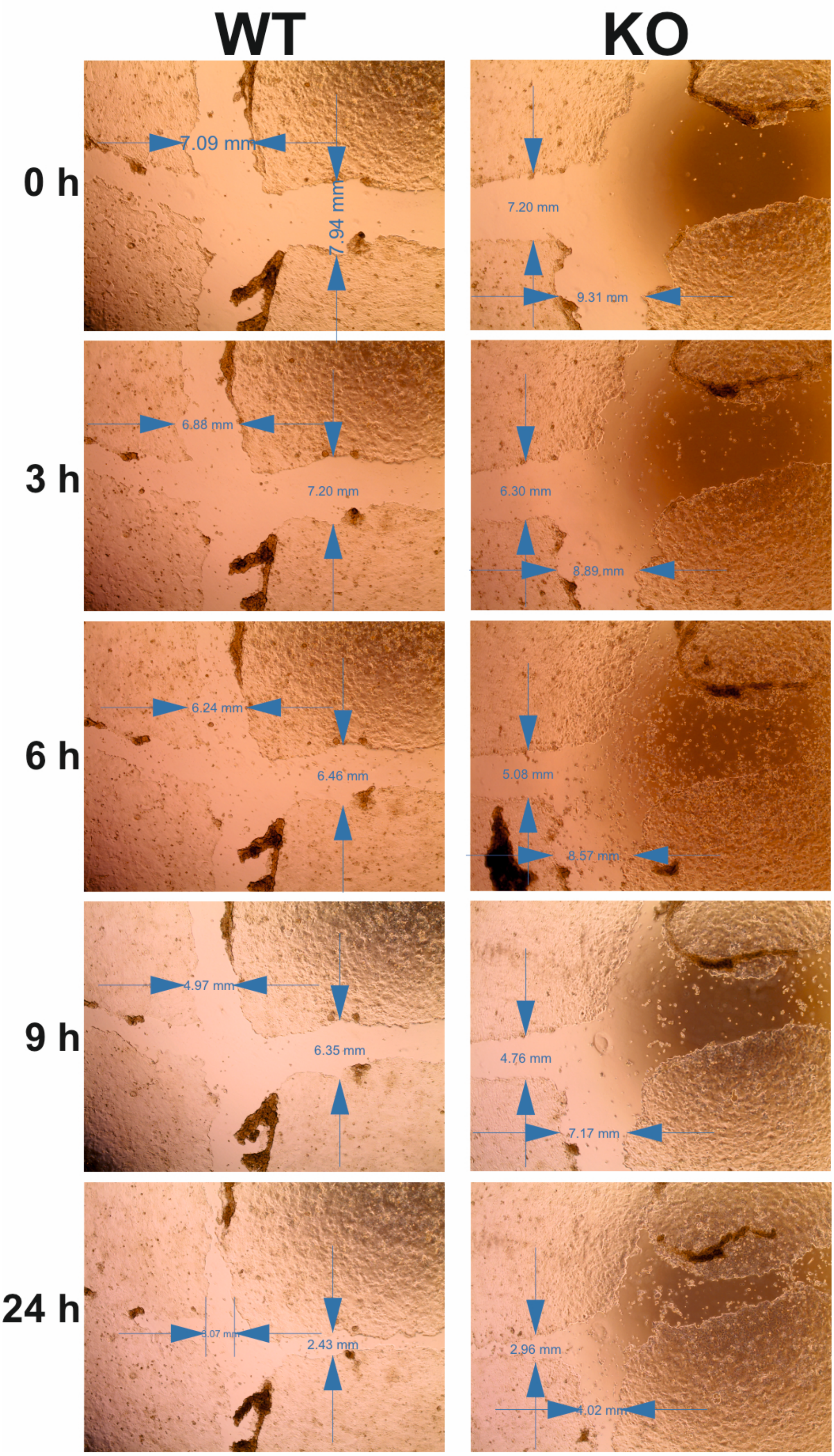

Figure S3. Scratch Assay. Microscopic images reveal the width of scratches and how much time has elapsed since they were made in wild-type (WT) and knockout (KO) Capan-1 cells. See the "Materials and Methods" section for more information.
